# Funnel plots, performance variation and the Myocardial Infarction National Audit Project 2003–2004

**DOI:** 10.1186/1471-2261-6-34

**Published:** 2006-08-02

**Authors:** Christopher P Gale, Anthony P Roberts, Phil D Batin, Alistair S Hall

**Affiliations:** 1 Academic Unit of Cardiovascular Medicine, G Floor, Jubilee Wing, The Yorkshire Heart Centre, The General Infirmary at Leeds, Great George Street, Leeds, West Yorkshire, LS1 3EX, UK; 2 2 Cardiothoracic Unit, The James Cook University Hospital, Marton Road, Middlesbrough, TS4 3BW, UK; 3 Department of Cardiology, Pinderfields General Hospital, Aberford Road, Wakefield, West Yorkshire, WF1 4DG, UK

## Abstract

**Background:**

Clinical governance requires health care professionals to improve standards of care and has resulted in comparison of clinical performance data. The Myocardial Infarction National Audit Project (a UK cardiology dataset) tabulates its performance. However funnel plots are the display method of choice for institutional comparison. We aimed to demonstrate that funnel plots may be derived from MINAP data and allow more meaningful interpretation of data.

**Methods:**

We examined the attainment of National Service Framework standards for all hospitals (n = 230) and all patients (n = 99,133) in the MINAP database between 1^st ^April 2003 and 31^st ^March 2004. We generated funnel plots (with control limits at 3 sigma) of Door to Needle and Call to Needle thrombolysis times, and the use of aspirin, beta-blockers and statins post myocardial infarction.

**Results:**

Only 87,427 patients fulfilled criteria for analysis of the use of secondary prevention drugs and 15,111 patients for analysis by Door to Needle and Call to Needle times (163 hospitals achieved the standards for Door to Needle times and 215 were within or above their control limits). One hundred and sixteen hospitals fell outside the 'within 25%' and 'more than 25%' standards for Call to Needle times, but 28 were below the lower control limits. Sixteen hospitals failed to reach the standards for aspirin usage post AMI and 24 remained below the lower control limits. Thirty hospitals were below the lower CL for beta-blocker usage and 49 outside the standard. Statin use was comparable.

**Conclusion:**

Funnel plots may be applied to a complex dataset and allow visual comparison of data derived from multiple health-care units. Variation is readily identified permitting units to appraise their practices so that effective quality improvement may take place.

## Background

Improving the quality of care in the National Health Service (NHS) by responding to variations in clinical processes and outcomes is an imperative required by the United Kingdom (UK) Government [1]. It has been prompted by incidents of failure of professional self-regulation, notably the Bristol and Shipman cases [2,3] and resulted in the collection of comparative data at all levels of healthcare provision. Though methods for using data to respond to variation are not established, [4] funnel plots are suggested as the display method of choice for institutional comparison [5].

Funnel plots are based on Statistical Process Control (SPC), a set of methods for ongoing improvement of systems, processes and outcomes [6-8]. Recently, comparative performance of UK cardiac surgeons has been disseminated using these plots [9,10] and they could be used to study comparative performance measures in other datasets such as the Myocardial Infarction National Audit Project (MINAP) registry (a UK cardiology dataset that characteristically represents its results as performance tables) [11]. We aimed to demonstrate that funnel plots may be derived from existing MINAP data and that they provide more meaningful interpretation of complex data.

## Methods

### Database

We studied all patients (and all hospitals in England who manage acute myocardial infarction (AMI)) who were entered into the MINAP database between 1^st ^April 2003 and 31^st ^March 2004. We tabulated the results of the MINAP database by the five variables reported in the MINAP Third Public Report [11], namely: Door to Needle Time (DTN), Call to Needle Time (CTN), and the use of aspirin, beta-blockers and HMG-CoA reductase inhibitors for secondary prevention (that is, drugs that reduce the risk of further AMIs). For the analysis we included all patients with an admission diagnosis of definite AMI that had no justified delay to treatment and received thrombolytic treatment. (Justified delays to treatment included hypertension, concern over risk of bleeding, delay in obtaining consent, non-diagnostic initial electrocardiograms, cardiac arrest, or insufficient information).

### Funnel plots

For each target we generated scatter plots of performance, as a percentage, against the number of cases reported (the denominator for the percentage). The mean hospital performance and exact binomial 3 sigma limits were calculated for all possible values for the number of cases and used to create a funnel plot using the method described by Spiegelhalter [11]. MINAP set absolute targets for achievement and we made funnel charts using 3 sigma limits around the target and around the mean. Only charts using a funnel based on the mean are presented (except for dtn30 for which both sets of limits are shown) as there was no substantial difference between methods for thrombolysis measures and for the secondary medication measures relatively few hospitals fell within the funnel's limits and many fell above the upper limit when the limits were set around the target. The absolute targets are also arbitrary as no one knows what the precise levels should be for these measures in real practice in the NHS. We also believe that most clinicians aim to be as good as their colleagues rather than seeking to meet a particular externally set clinical target. The measures used are process rather than outcome measures. The three funnel plots for use of secondary prevention medications show variation which seems related to volume of cases – larger volumes seem to relate to lower achievement. Is there a systematic reason why care, or recording of care, is more difficult in larger units? Although the clinical processes measured do not vary solely by chance, comparing the variation in results to chance helps us see where differences are important enough to investigate. We consider this is a good reason for showing the results in a scatter as this variability is very difficult to see in a table.

### Special-cause and common-cause variation

When the performance of clinical units is compared, one might expect process measures to not necessarily display variability consistent with chance, as there are likely to be systematic reasons for differences. Variation may be attributable to either 'common-cause variation' or 'special-cause variation'. We considered that units displayed 'special cause variation' when their performance fell beyond the limit lines of the funnel plot and that they were located there due to the presence of systematic influences [12].

We considered that units displayed 'common-cause variation' when their performance fell within the limit lines of the funnel plot indicating that their performance varied only by an amount consistent with random chance. It would be expected that the patient factors that influence the clinical decisions being measured at unit level would be likely to present randomly to units across the UK and that decision-making might therefore be expected to vary by this amount if no systematic differences in the decision-making processes and thresholds exists between units.

Common-cause variation (because it is linked to chance), is greatest when numbers of patients are small (left of funnel plot) and reduces as numbers of patients per unit increases. While being within the funnel plot's limits does not exclude the possibility of more moderate or opposing systematic influences being present, the most likely explanation for the variation seen for units within the limit lines of the funnel plot is that it results from common-cause variation. This is background noise that is a feature of the process itself [13]. It may reflect local variances in hospital-specific practices and policies such as day-to-day variations in staffing levels or marginal differences in transit times (for geographical reasons).

### Comparison

We used the Planning and Performance Framework (2003–6) standards to define achievement as those 'reaching the goal', 'within 25% of the goal' and 'more than 25% from the goal'. Attainment of these standards is used in the derivation of performance tables for the annual MINAP reports. Achievement goals are different for thrombolytic treatment (DTN and CTN) and secondary prevention (aspirin, beta-blockers and HMG-CoA reductase inhibitors). For the DTN standard, 75% of patients were required to receive thrombolytic treatment within 30 minutes whereas for the CTN standard 48% of patients were required to receive thrombolytic treatment within 60 minutes in 2003–4. The standard for secondary prevention treatments was that 80% of patients discharged from hospital should receive aspirin, beta-blockers and HMG-CoA reductase inhibitors. We used these definitions to generate results for our dataset and to compare them with the number of hospitals above, below or within the control limits as calculated by our funnel plots. The numbers of hospitals that had fewer than 20 cases, but met the MINAP analysis criteria were also recorded.

## Results

There were 99,133 patients in the MINAP database, which covered between 225 and 230 hospitals for the five variables. Only 87,427 fulfilled the inclusion criteria for analysis of the use of secondary prevention drugs (final diagnosis of AMI) and 15,111 for analysis by DTN and CTN times (as only ST-Elevation AMI patients are eligible for thrombolysis). For the five MINAP output variables (CTN, DTN, use of aspirin, beta-blockers and HMG-CoA reductase inhibitors) between 8 and 25 hospitals were excluded from the MINAP performance tabulation, but were included in the SPC analysis.

**Figure 1:**
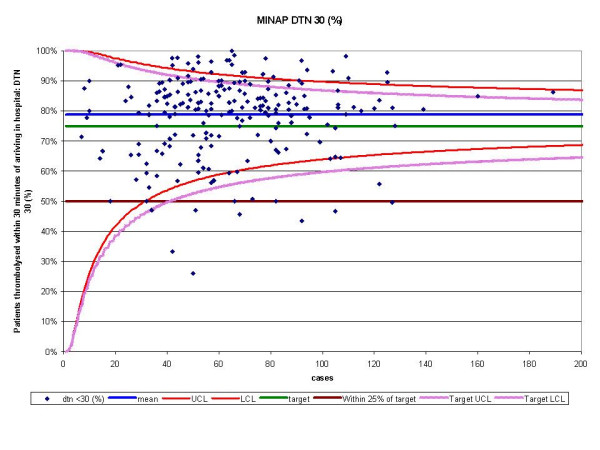
Funnel plot for percentage of patients thrombolysed within 30 minutes of arriving in hospital (DTN 30). Data abstracted from the MINAP Third Public Report.[11] DTN = door to needle time, LCL = lower control limit, UCL = upper control limit, target = National Service Framework for coronary heart disease goal of 75%. Funnel plot with control limits for both mean performance and target performance. In this case the number of hospitals identified is similar.

**Figure 2:**
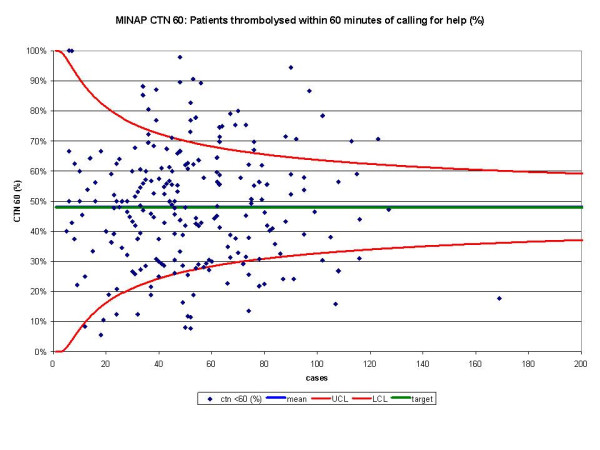
Funnel plot for percentage of patients thrombolysed within 60 minutes of calling for help (CTN 60). Data abstracted from the MINAP Third Public Report.[11] CTN = call to needle time, LCL = lower control limit, UCL = upper control limit, target = National Service Framework for coronary heart disease goal of 48%. Mean and target performance coincide at 48%.

**Figure 3:**
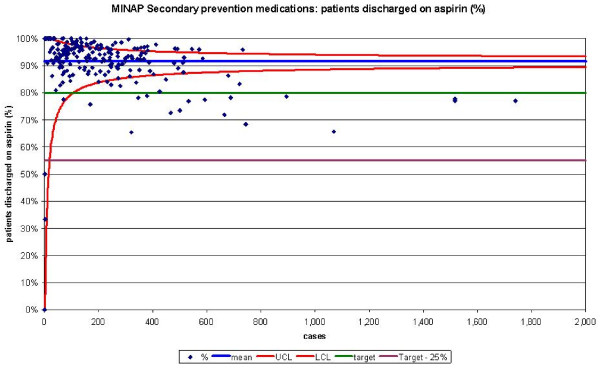
Funnel plot for percentage of patients receiving aspirin as secondary prevention medication on discharge from hospital. Data abstracted from the MINAP Third Public Report.[11] LCL = lower control limit, UCL = upper control limit, target = National Service Framework for coronary heart disease goal of 80%, target 25% = more than 25% from the goal.

**Figure 4:**
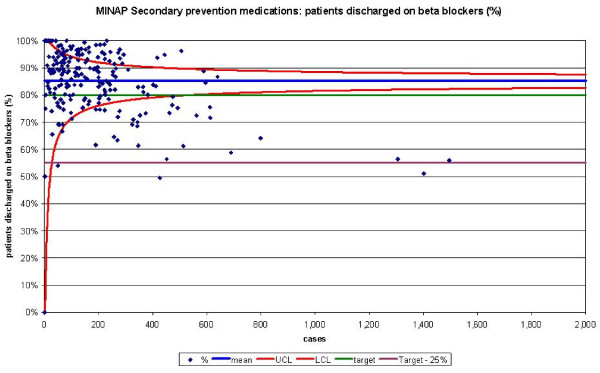
Funnel plot for percentage of patients receiving a beta blocker as secondary prevention medication on discharge from hospital. Data abstracted from the MINAP Third Public Report.[11] LCL = lower control limit, UCL = upper control limit, target = National Service Framework for coronary heart disease goal of 80%, target 25% = more than 25% from the goal.

**Figure 5:**
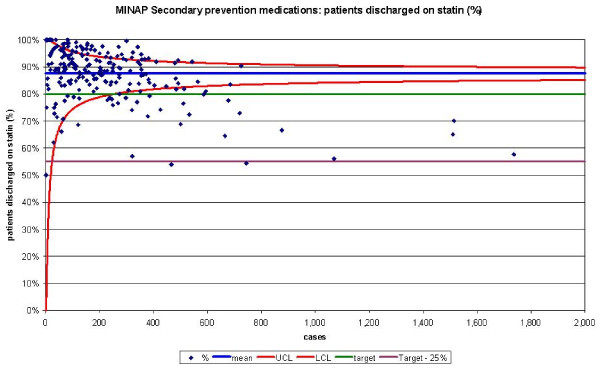
Funnel plot for percentage of patients receiving a HMG-CoA reductase inhibitor as secondary prevention medication on discharge from hospital. Data abstracted from the MINAP Third Public Report.[11] Statin = HMG-CoA reductase inhibitor, LCL = lower control limit, UCL = upper control limit, target = National Service Framework for coronary heart disease goal of 80%, target 25% = more than 25% from the goal.

The funnel plots for thrombolytic treatment goals demonstrated a wide dispersion of process data and nearly as many hospitals were above the control limits as below (figures 1 and 2). The funnel plots for secondary prevention showed a similar amount of dispersion beyond control limits (figures 3, 4 and 5).

For DTN, we identified 163 hospitals that achieved the performance standards, but 215 hospitals were found within the funnel or above the upper control limit (table 1). For CTN, 116 hospitals fell outside the 'within 25%' and 'more than 25%' performance standards, whereas using SPC we only identified 28 below the lower control limit.

**Table 1:** Comparison of the numbers of hospitals achieving NSF goals and the number inside and outside 3 sigma limits.

	**DTN**	**CTN**	**Aspirin**	**Beta-blocker**	**Statin**
**Analysis by attainment of NSF targets**					

Reaching the goal	163	102	191	151	180
Within 25% of the goal	51	0	16	46	24
More than 25% from the goal	8	116	0	3	2
Less than 20 cases	8	12	20	25	20
Total	230	230	227	225	226
**Analysis by funnel plot**					
					
Above UCL	33	32	40	43	34
Within limits	182	170	187	152	164
Below LCL	15	28*	24	30	31
Total	230	230	227	225	226
**Discrepancy between table and funnel plot assessment of performance**					
					
Within target and below LCL	0	0	8	0	5
Within 25% of target but below LCL	8	28*	16	27	24
Beyond 25% of target and below LCL	7	0	0	3	2
Within target but above UCL	27	32	37	44	35
Total discrepancy	42	60	61	74	66

DTN = door to needle time, CTN = call to needle time, Statin = HMG-CoA reductase inhibitor, NSF = National Service Framework for Coronary Heart Disease.

*The 'within 25% of target' did not apply to CTN 60. In total, 108 hospitals were above the 48% target and 122 below it, of which 28 were also below the LCL.

When we compared the numbers of hospitals achieving performance standards for the medication targets with funnel plot depictions, we found that despite them being 'within 25%' of their attainment standards, many hospitals were below the lower control limits. Sixteen hospitals failed to reach the performance standards for aspirin usage post AMI, but when analysed by SPC these 16 and a further 8 hospitals remained below the lower control limit. For beta-blockers usage, 30 hospitals were below the lower control limit and 49 outside the achievement standards. HMG-CoA reductase inhibitor use was comparable from SPC analysis (31 below the lower control limit) and performance standards (and 26 not achieving the performance standard).

For the five MINAP output variables, between 26 and 43 hospitals where found to lie above their respective upper control limits (table 1).

## Discussion

Other statistical methods for health-care surveillance exist [14-16], but funnel plots offer readily interpretable plots of multiple unit comparisons that allow for sample size, using a scale that is intuitive for clinicians to use. Funnel plots permit SPC assessment to be applied to a complex dataset using crude (not case-mix adjusted) comparison of outcome data derived from multiple healthcare units. The analysis is not restricted by number of cases per unit. Common-cause and special-cause variation can be readily visualised (through the identification of units outside of the funnel) and it permits each unit to appraise their local practices e.g. low use of statins at discharge.

### Special-cause and common-cause variation

Special-cause variation was identified in the MINAP funnel plots. The thrombolysis funnel plots revealed a wide dispersion of data suggesting a single consistent process of care was not occurring across the hospitals, but that multiple processes were producing the measured outcome. This is not surprising as the call to door process (carried out by ambulance services) is a different process to door to needle (carried out by hospitals). Though the secondary prevention funnel plots conformed to the control limits, outliers were readily identified below the lower control limits suggesting under achievement. A number of hospitals lay more than 3 sigma above the mean signifying either good clinical practice or favourable systematic biases in the collection and submission of data. Just as areas for improvement can be identified in some units, unit processes that have contributed to high attainment of National targets can be used as examples of 'good practice' that might be reproduced in other hospitals. Where the number of cases is smaller than expected (perhaps because of unusually high excepting of patients from the measure), the relative position along the x axis compared with other similar units is useful, although MINAP use codes rather than hospital names as identifiers.

We noted that for some hospitals, high numbers of patients were receiving secondary prevention medications but this did not correspond to the numbers of patients receiving thrombolysis. This is because analysis for secondary prevention is performed for all patients having AMI (whether ST segment elevation, non-ST elevation, threatened AMI or unconfirmed AMI). Alternatively, some units may have focused resources on one target (e.g. by provision of a 24 hour thrombolysis-nurse service) in contrast with a second unit focussing on an alternate target (e.g. by provision and adherence to an acute coronary syndrome care pathway). Other explanations include local variations in case-mix as might result from alternate definition of AMI and availability, use and types of cardiac enzyme markers [17].

The funnel plots for use of secondary prevention medications show variation which is related to volume of cases (larger volumes correspond to lower achievement). This may be because the recording of care, might be more difficult in larger hospitals and/or the denominators for some may be incorrect (some hospitals may have included patients who wouldn't have qualified clinically for secondary prevention drugs). If the funnel plots were labelled with the hospitals' names, each could then check they have denominators of the size expected.

### Performance tabulation

As expected, there was a discrepancy in counts across the hospitals between SPC methodology and performance tabulation [18]. Up to a third of counts may be scored differently when SPC methodology is used in preference to performance tabulation. In part, this is due to the construction of control limits around mean performance rather than the absolute performance target. It also reflects the ability of SPC to include all hospitals in the analysis and to more precisely demonstrate evidence of performance based on sample size. Small hospitals are subject to greater sampling error in estimates of performance and an observed failure to meet a performance standard may occur when appropriate procedures are being followed (if by chance the small numbers of patients being treated had an unusual profile). The lack of an upper limit in the performance table contributes between half and two thirds of the discrepancy.

Performance tables polarise results. They create a state of 'sanctuary' in hospitals above the cut-off and a state of 'alarm' in those below it. Special-cause variation may be overlooked because results are not considered in the context of control limits (that may be used as a guide to when it is uneconomic to look for assignable causes). SPC allows those hospitals with smaller numbers of patients to have a greater acceptable variance avoiding the need for them to be excluded. Moreover, the use of categories of performance (e.g. the 'within 25% category') may lead to inaccurate hospital performance estimates. They may overestimate performance because despite some hospitals being 'within 25%' of their attainment goals, many were found to be below the lower control limits. This occurs in hospitals with a greater number of cases (figure 4 and 5) and highlights the fact that the use of pre-set targets (that are independent of case volume) is problematic.

Because performance tables overlook special-cause variation, they may misdirect attention to less important common-cause variation. The secondary prevention tabulation of the performance standards suggests near universal compliance with the targets, which although reassuring, may incline hospitals towards thinking no further action is needed. It may also mask excessive treatment of some patients (e.g. the elderly, for which the evidence base is less convincing). Funnel plots identify those hospitals that are under performing and visually suggest that improvements may still be made in adequately performing hospitals. Multi-level modelling would enable a unit's progress to be plotted over time and identify those hospitals that are 'coasting' at just above the target [19]. Although the clinical processes measured do not vary solely by chance, comparing the variation in results to chance helps us see where differences are important enough to investigate. We consider this is a good reason for showing the results in a scatter as this variability is difficult to see in a table.

### Limitations of SPC

Like all performance measurement and analysis methods, SPC funnel charts may produce both false negative and positive results. Performing within limits does not guarantee that a unit may not be underperforming though this may either be too slight to detect or masked by other factors. Being outside a control limit is not always abnormal (special cause) either. Of the 230 hospitals, 2 or 3 may be outside the 3 sigma (99.8%) limits by chance alone. Despite such false negative and positive results, it is important to remember that truly detecting a 'special cause' encourages further investigation at the level of the individual hospital (and not missing this opportunity). Similarly funnel plots help prevent investigation of an outcome resulting from a common-cause as if it were a special-cause.

### Improvement activity

Credibly using data to raise local awareness among health care staff of the need for improvements in processes is vital when there is under-achievement in clinical performance. In the event that units believe the implications of the funnel plots to be clinically invalid, then attention might rather focus on possible anomalies in the process of data-gathering and reporting at that unit as compared to others.

## Conclusion

The Myocardial Infarction National Audit Project is at the forefront of the clinically-led registries, with the collection of consistent, comprehensive and accurate clinical information about a specific group of patients. It represents the most robustly collected data of its type and as such is a key pioneering project in which different methods of analysis can be tested. We have demonstrated that the extensive clinical output data may be visualised for direct comparison across multiple units using funnel plots. They avoid the worst of the polarisation of results associated with the 'ticks and crosses' in performance tables and allow the identification of potential areas of achievement that warrant further attention to permit continuing improvement in the quality of care offered to patients. The routine publication of funnel plots summarising cardiology performance attainments will encourage the understanding, reflection, processes analysis and subsequent improvements in health-care at all levels of policy, planning and actual delivery.

## Competing interests

The author(s) declare that they have no competing interests.

## Authors' contributions

CG researched and wrote the manuscript. AR performed the statistics and wrote the article. PB and AH reviewed, wrote and critically appraised the article. All authors read and approved the final manuscript.

## Pre-publication history

The pre-publication history for this paper can be accessed here:


